# Toothpaste Abrasion and Abrasive Particle Content: Correlating High-Resolution Profilometric Analysis with Relative Dentin Abrasivity (RDA)

**DOI:** 10.3390/dj11030079

**Published:** 2023-03-12

**Authors:** Joachim Enax, Frederic Meyer, Erik Schulze zur Wiesche, Ines Christin Fuhrmann, Helge-Otto Fabritius

**Affiliations:** 1 Research Department, Dr. Kurt Wolff GmbH & Co. KG, Johanneswerkstr. 34-36, 33611 Bielefeld, Germany; 2 Bionics and Materials Development, Department Lippstadt 1, Hamm-Lippstadt University of Applied Sciences, 59063 Hamm, Germany

**Keywords:** abrasion, polymethyl methacrylate, profilometry, RDA, scanning electron microscopy, silica, toothpaste

## Abstract

In this in vitro study, the influence of the concentration of abrasive particles on the abrasivity of toothpastes was investigated using laser scan profilometry on polymethyl methacrylate (PMMA) surfaces with the aim of providing an alternative method to developers for screening of new toothpaste formulations. PMMA plates were tested in a toothbrush simulator with distilled water and four model toothpastes with increasing content of hydrated silica (2.5, 5.0, 7.5, 10.0 wt%). The viscosity of the model toothpaste formulations was kept constant by means of varying the content of sodium carboxymethyl cellulose and water. The brushed surfaces were evaluated using laser scan profilometry at micrometer-scale resolutions, and the total volume of the introduced scratches was calculated along with the roughness parameters Ra, Rz and Rv. RDA measurements commissioned for the same toothpaste formulations were used to analyze the correlation between results obtained with the different methods. The same experimental procedure was applied to five commercially available toothpastes, and the results were evaluated against our model system. In addition, we characterize abrasive hydrated silica and discuss their effects on PMMA-sample surfaces. The results show that the abrasiveness of a model toothpaste increases with the weight percentage of hydrated silica. Increasing roughness parameter and volume loss values show good correlation with the likewise increasing corresponding RDA values for all model toothpastes, as well as commercial toothpastes without ingredients that can damage the used substrate PMMA. From our results, we deduce an abrasion classification that corresponds to the RDA classification established for marketed toothpastes.

## 1. Introduction

Domestic dental care, at least twice a day, has been shown to be crucial for the prevention of tooth decay and periodontal disease. These are the world’s most common diseases, which are caused mainly by the presence of microbial biofilms (plaque) [1,2]. Related studies have demonstrated that two minutes of brushing resulted in a significant plaque reduction of more than 40% [3]. The cleaning performance is influenced mainly by the toothbrush and the use of toothpastes [4]. Abrasive cleaning particles are present in almost all commercially available toothpaste formulations worldwide. Their function is to help remove plaque and stains while causing only negligible damage to tooth structures or gums [5,6,7,8]. The most commonly used abrasive today is particulate hydrated silica, which is present in toothpastes at varying concentrations [5,8].

In order to determine the abrasiveness of a toothpaste formulation, different methods (copper abrasion tests, radioactive enamel abrasion, profilometry, etc.) are used [9]. Currently, the most common method is the measurement of the relative dentin abrasion (RDA) according to the radiotracer (Rt) method and the profilometry equivalent method (RDA-PE). Due to the complexity of the procedure, RDA determinations are very time-consuming and cost-intensive. Furthermore, examinations at different institutes have led to major fluctuations without a systematic trend in the measured values [9], which limits the comparability of the results [10]. These deviations can be attributed on the one hand to differences in the individual composition and structure of the used teeth and on the other hand to the challenge of producing clean, uniform sample surfaces from natural teeth [11]. Different reference abrasives such as silica and calcium pyrophosphate used for the test further complicate the comparison [10].

The aforementioned weaknesses of the RDA method make it difficult for developers of oral care products to monitor the abrasive properties of new toothpaste formulations at any selected time. Therefore, a method that can be performed with comparatively little effort in the lab is desirable, which enables the quick adjustment of formulations to specific abrasivity requirements which are necessary for, e.g., whitening or sensitive products during the development process. Therefore, the aim of this study was to develop an alternative and complementary standardized method based on profilometric analysis and to investigate how well it correlates with the existing standardized abrasivity tests (RDA, RDA-PE). For this, we tested how increasing concentrations of hydrated silica (2.5, 5.0, 7.5%, 10.0 wt%) affect the abrasion behavior of a model toothpaste and compare the profilometric results with corresponding RDA values. To exclude influences of inhomogeneous substrates, the brushing simulator tests for our four model toothpastes were carried out on polymethyl methacrylate (PMMA) plates [12,13]. PMMA has a hardness comparable to dentine [12]. Furthermore, the use of PMMA plates standardizes the test setup, since there are no biologically caused deviations that have an impact on the measured values.

## 2. Materials and Methods

### 2.1. Model Toothpastes

Four toothpaste formulations with different contents of abrasive hydrated silica particles (2.5, 5.0, 7.5 and 10.0 wt%) were prepared (see Table 1). The formulation also contains a preservative system, humectants, components for cleaning and foaming and viscosity-modifying components. The ingredients were mixed using a STEPHAN universal machine UMC 5 mixer (Stephan Food Service Equipment GmbH, Hameln, Germany) under ambient conditions. The formulations were adjusted to have a toothpaste-like consistency. The viscosities were adjusted to about 50 Pa·s (variation < 15%) by varying the concentration of sodium carboxymethyl cellulose and water in dependence of the hydrated silica content (see Table 1). The formulations were allowed to rest for one week at 40 °C. Their final dynamic viscosity was determined by using a HAAKE RheoStress 1 rotational rheometer (Thermo Fisher Scientific, Waltham, MA, USA) with a plate–plate measuring geometry (PP60Ti, 60 mm diameter). The measurements (200 data points for every sample) were started in CR mode by heating the samples (23 °C for two minutes) with a gap size of 0.50 mm. All four toothpastes were able to form a strand that did not sink into the bristle bundles when applied to a toothbrush by using a tube, even after a few minutes. Before sending aliquots of the model toothpastes out for RDA determination, their pH was measured using a Mettler Toledo Seven Compact pH meter (Mettler Toledo GmbH, Giessen, Germany) (Table 2).

### 2.2. Abrasion Tests

**Figure 1:**
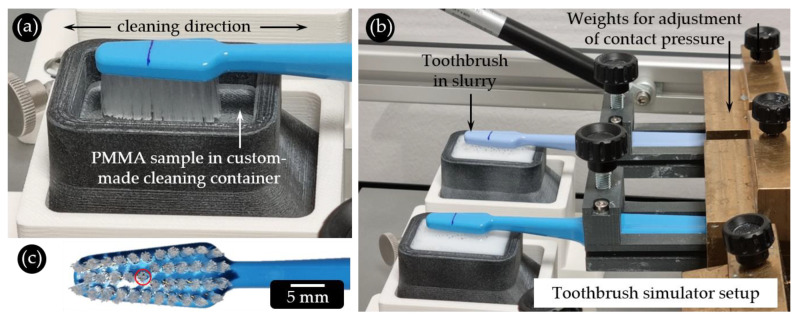
(**a**) Abrasion test setup. The bristles of the mounted toothbrush are parallel to the sample substrate in a custom-made container. (**b**) Overview of the brushing simulator during testing. (**c**) Toothbrush head of the TePe Select Medium manual toothbrush used for the cleaning tests. The toothbrush tuft to be examined is encircled in red.

All abrasion tests were performed in the Dr. Kurt Wolff Oral Care Laboratories, Bielefeld, Germany, using a ZM-3.8 toothbrush simulator (SD Mechatronik GmbH, Feldkirchen-Westerham, Germany) equipped with eight slots (Figure 1).

As a substrate for the abrasion tests, extruded PMMA plates (Kahmann & Ellerbrock GmbH & Co. KG, Bielefeld, Germany) with a standardized and reproducible surface quality were acquired. The plates had dimensions of 37 × 27 × 3 mm, a density of 1.19 kg/m^3^ and a transmission of 92%. The scratch protection foils on both sides of the PMMA plates were removed only shortly before starting the experiment to avoid pre-damaging the surfaces.

TePe Select Medium manual toothbrushes (TePe Munhygienprodukter AB, Malmö, Sweden) were used for the tests. This toothbrush has 39 tufts (11 rows with 2–4 tufts per row) with approx. 35 filaments. The filaments all have the same length, creating a planar cleaning surface (Figure 1a,c). Each toothbrush was mounted in the simulator so that the bristle surface was plane-parallel to the sample surface. The contact pressure of 1.5 N was calibrated using a mechanical spring balance (Kern & Sohn GmbH, Balingen, Germany) and set by moving the weight attached to each brushing arm in the right position (Figure 1b). The abrasive potential of the toothbrush itself was tested by performing brushing tests on PMMA in distilled water, which were used as reference against the toothpastes.

In addition to the four model toothpastes, five commercial toothpastes were included in the abrasion tests: elmex^®^ Kariesschutz-Zahnpasta; elmex^®^ Kinder-Zahnpasta (1000 ppm fluoride as amine fluoride) (both CP GABA GmbH, Hamburg, Germany); Bioniq^®^ Repair-Zahncreme Plus; Bioniq^®^ Repair-Zahncreme; and Kinder Karex Zahnpasta (all Dr. Kurt Wolff GmbH & Co. KG, Bielefeld, Germany). From all toothpastes, a slurry was mixed (25 g toothpaste with 40 mL distilled water [10]) and mechanically stirred on a magnetic plate (300 rpm, 5 min) before being poured into the sample chambers (Figure 1b).

Using this setup, tooth brushing was simulated by cleaning with a linear movement pattern in the y-direction. The travel distance of the toothbrush was 5 mm in each direction (per 1 cycle). For each sample, 6000 cleaning cycles [10] were performed at a cleaning speed of approx. 20 mm/s, which resulted in a total cleaning time of 57 min.

After completion of each toothbrush simulator test, the samples were removed from the holders and cleaned 3 times with distilled water. Then, they were wiped with cotton in the direction of brushing without applying pressure. In between, short ultrasonic cleaning steps (1 min) were performed in distilled water. Finally, the samples were rinsed thoroughly with distilled water from a spray bottle and further dried in air.

### 2.3. Profilometric Recording of Abrasion

**Figure 2:**
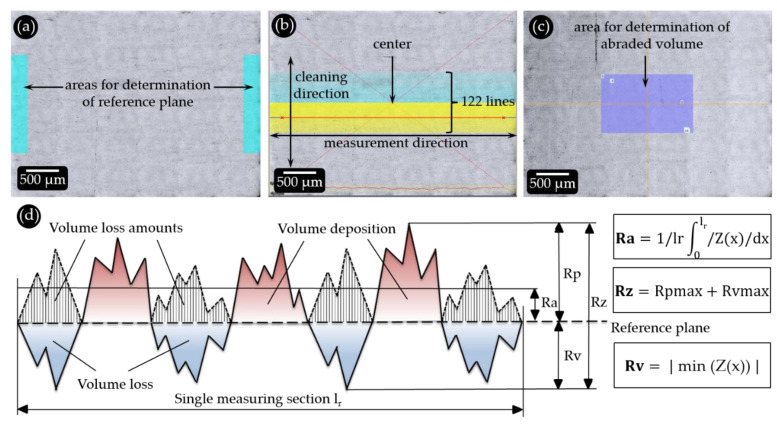
(**a**) Defining the reference surfaces (turquoise areas). (**b**) A total of 122 measurement lines at intervals of 10 pixels at a 90° angle to the cleaning direction. (**c**) Selected area for measuring volume loss (purple area). (**d**) Schematic depiction of how the roughness parameters Ra, Rz and Rv are defined, including the formulae used for their calculation, according to [14].

All profilometric parameters were obtained using a Keyence VK-X1100 laser scanning microscope (Higashi-Nakajima, Japan), and the included viewer software package (version 1.4.0.234). The images were recorded with a 20× objective. This allowed detection of even the finest scratches in the micrometer range induced by the toothbrush bristles on the sample surfaces. Each image consisted of 36 (6 rows of 6) individual images that were stitched together. The resulting imaged area measured approx. 3.8 × 2.8 mm (Figure 2). This area corresponds to the area touched by a single toothbrush tuft during the abrasion tests. For profilometric analysis, the area covered by the fifth tuft from the central row was chosen (as viewed from the tip of the toothbrush, see Figure 1c).

### 2.4. Profilometric Analysis of Abrasion

The material loss caused by the tooth-brushing tests was analyzed using the MultiFileAnalyzer software (Version 2.2.0.93, Keyence (Higashi-Nakajima, Japan)). Two rectangular reference areas (250 µm width × 1333 µm height) were determined on the left and right outer margin of each image, the average height values of which define the reference plane (Figure 2a). To obtain all relevant parameters, both the line roughness of cross sections (multiple line roughness, Figure 2b) and the volume loss (Figure 2c) were quantified.

Three roughness parameters were determined using the multi-line roughness tool of the software [14]. The arithmetic mean roughness value Ra is the average deviation of the profile from the reference surface, which is calculated from the amounts of the profile values over all the individual measured sections (Figure 2d). The average roughness depth Rz is the arithmetic mean of the individual roughness depths of all measured sections (Figure 2d). The individual roughness depth is the greatest distance from the lowest to the highest point within an individual measured section [14]. The maximum profile depth Rv indicates the maximum vertical distance between the reference surface and the deepest point within a single measurement section (Figure 2d). The volume loss is determined as the volume of all scratches below the reference surface in a defined area of the sample (Figure 2c).

For each model toothpaste and commercial product, three samples were tested. After determination of the center of the recording, 122 parallel profile measurement lines were defined around the center at a distance of 10 pixels and at an orthogonal angle to the cleaning direction (Figure 2b). For quantification of the abraded volumes, an area of 1400 µm width × 890 µm height was defined within the previously selected measurement area for the multi-line roughness (Figure 2c).

### 2.5. SEM Analysis of Hydrated Silica Particles and Abrasion Test Samples

**Figure 3:**
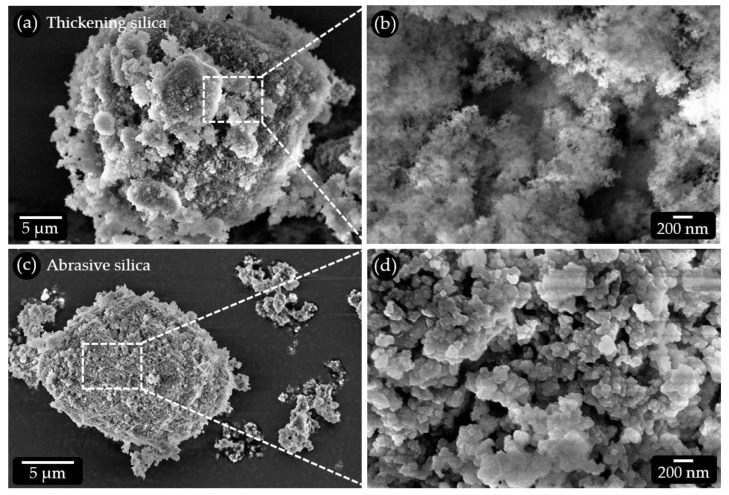
SEM micrographs of the hydrated silica particle types used for the model toothpastes. (**a**) Thickening hydrated silica particles show a very finely structured surface. They consist of (**b**) nanoscopic coherent particles creating a high porosity. (**c**) Abrasive hydrated silica particles are more compact and consist of (**d**) about 200 nm large, roughly polyhedral particles with sharp edges that form clusters.

For scanning electron microscopic (SEM) analysis of the abrasive and thickening hydrated silica particles (Table 1, Figure 3), a small amount of each of the powders was applied to self-adhesive carbon pads adhering to standard aluminum holders using a spatula. Non-adherent particles were removed by gentle tapping. The samples were coated with an approx. 5 nm thick layer of platinum/palladium (80:20) in a sputtering system (Q150T ES, Quorum TECHNOLOGIES LTD, UK) under rotation at a tilt angle of about 15°. The samples were examined in a Zeiss Sigma SEM with a Gemini column (Zeiss, Oberkochen, Germany). Images were recorded at an acceleration voltage of 5 kV using a 30 µm aperture and an in-lens detector at small working distances (<5 mm) for high resolution.

### 2.6. Determination of Radioactive/Relative Dentine Abrasion (RDA)

Blinded RDA measurements of our four model toothpaste formulations with different contents of particulate abrasive hydrated silica were commissioned from Therametric Technologies, Inc., (IN 46,060 Noblesville, IN, USA). In addition, RDA measurements of the five commercial products (see Section 2.2 and Table 3), all of which use hydrated silica as the cleaning agent, were commissioned from the same laboratory to obtain absolutely comparable data for the verification of our cleaning tests. The relative dentine abrasion of the nine toothpastes was determined according to ISO 11609 [10] by testing eight polymethyl methacrylate-embedded human dentine samples in a V-8 cross-brush machine. The toothpaste slurries were prepared according to ISO 11609 [10] by mixing 25 g of toothpaste with 40 mL of deionized water. Eight measurements were taken for each toothpaste, and the mean and the standard deviation were determined.

## 3. Results and Discussion

### 3.1. Model Toothpastes and Substrate Material

Our model toothpaste formulations were designed to contain typical ingredients that are also used in commercial products, except for specific active ingredients (e.g. remineralization or antibacterial agents) (Table 1). The main goal of this in vitro study was to obtain data on the abrasivity of toothpastes depending on the content of abrasive particles by using laser scan microscopy in conjunction with profilometry, i.e., a quantitative analysis of the scratch profiles created over a defined time by toothbrush filaments on a standardized sample surface. Scratches on the PMMA surface can result from several sources: the toothbrush filaments themselves, the abrasive particles and other solid components contained in a toothpaste formulation. In addition, the consistency of the tested toothpaste can influence the different sources of abrasive damage by favoring different damage mechanisms. To obtain results in which the measured effect is related to the abrasive particles, the influence of the other parameters should be reduced. To address this, we pursued two strategies. Firstly, we adjusted the composition of our model toothpastes to obtain similar physical and chemical properties such as viscosity and pH. This equalizes influences of the brushing procedure itself. Secondly, we tested the influence of the toothbrush alone by performing abrasion tests with distilled water that can be used as a reference against the toothpastes.

To minimize the influence of viscosity on the brushing process, all model toothpastes had to fulfill the criterion of being applicable to a toothbrush in the form of strands without sinking in between the individual bristle filaments [15]. A dynamic shear viscosity of 50.0 Pa·s was determined as the target value for the model toothpastes. The viscosity increases with increasing abrasive silica content. This had to be compensated by decreasing the content of water and the concentration of one of the two thickening agents used. We chose to vary the sodium carboxymethyl cellulose, since the thickening hydrated silica used are of particulate nature (Figure 3a,b). It cannot be excluded that they may contribute to the abrasive effect. Therefore, their content was kept constant to minimize errors. The water facilitates the swelling and hydration of the sodium carboxymethyl cellulose, which is crucial for holding the liquid and the abrasive together [16]. This renders an exact adjustment of the viscosities difficult since they change over time due to swelling and hydration. We therefore allowed a maximal deviation of 15.0%. All formulations showed an average dynamic shear viscosity between 45.1 Pa·s and 56.7 Pa·s (Table 2).

The RDA-profilometry equivalent (RDA-PE) method is an established method for the determination of toothpaste abrasivity, and the method yields linearly correlated and proportional results compared to the radiotracer-based standard RDA-method [10,17,18]. Both RDA and RDA-PE use polished dentine slabs as substrate materials. The abrasiveness of a toothpaste is determined by either quantifying the amount of removed material versus a control abrasive (RDA) or quantification of the mean depth of abrasion below a reference plane, which is determined by masking tape on the dentine surface versus a control abrasive (RDA-PE) [17,18]. Obtaining comparable results for abrasion using profilometric methods requires a very smooth and reproducible surface quality [12]. The combination of PMMA surfaces with laser-scan profilometry enables the characterization of scratch patterns with resolutions in the sub-micrometer range. This allows for conclusions to be drawn about the abrasion mechanisms down to the level of the interaction of individual abrasive particles with the sample surface. Human or bovine dentine required for RDA and RDA-PE measurements [10] has been shown to be polishable to very flat surfaces [19]. However, the exposed lumina of the inherently present dentine tubules will always create a defective sample surface. This makes it difficult to detect scratches from toothbrushing in a quantitative way using profilometry, especially if toothpastes with low abrasivity are tested. In addition, the toothpaste will be pressed into the tubules during brushing and would be difficult to remove thoroughly before profilometric analysis. Moreover, the active ingredients present in toothpastes may unfold their effects on the dentine during the rather long testing times. This could result in further alterations of the sample surface. Although using PMMA as the specimen material has its drawbacks (e.g., different chemical composition compared to the tooth mineral), standardized experimental conditions are created. Biologically induced deviations are excluded, and time-consuming sample preparation is not necessary [10,20]. Furthermore, many different formulations can be tested cost-effectively due to easy availability and low sample material costs.

The pH values of our model toothpastes were considered to be of only minor relevance for our abrasion tests. With PMMA as the substrate material, we did not expect any erosion effects. However, pH-induced erosion can be relevant when brushing tooth material, as it occurs in the RDA tests. All model toothpastes had an almost neutral pH between 6.3 and 6.5 (Table 2). The pH values of the slurries (25 g toothpaste in 40 mL distilled water) were between 6.8 and 6.9 (Table 2). The critical pH value for dentine is between 6.2 and 6.4 [21] and, thus, below the values measured for the toothpaste slurries. It can be expected that the slurry used for the determination of the RDA values did not have an erosive effect on the dentine and, thus, no effect on the results.

### 3.2. Microstructure of the Used Hydrated Silica Particle Qualities

To elucidate the abrasive potential of the hydrated silica particles included in our model toothpaste formulations, both qualities used (see Table 1) were subjected to SEM analysis (Figure 3). The average particle size of the thickening hydrated silica quality is about 20 µm (Figure 3a). This is slightly larger than the size of the abrasive hydrated silica quality with an average particle size of about 14 µm (Figure 3c). At high magnification, the thickening hydrated silica particles show a very loose structure consisting of aggregated nanoscopic particles with a high intrinsic porosity (Figure 3b). These particle aggregates do not form a compact mass but leave additional larger pores on the higher structural levels. Due to the very small size of the building blocks, mechanical forces acting on these particles can be expected to lead to disintegration. The probability that they induce scratches on tooth or PMMA surfaces is low. In addition, the high porosity enables a high uptake of liquid during swelling. The liquid would act as a lubricant when these particles are forcibly moved on a surface. This is supported by the relatively high DOA absorption number (oil absorption) of 2.50 mL/g, as specified by the manufacturer. The DOA absorption number provides an indication of the void volume. High numbers are associated with a low specific surface area, a large average pore size and a high pore volume [22]. The abrasive hydrated silica particles consist of about 100–200 nm sized building blocks with roughly polyhedral shapes and sharp edges. They are densely packed, resulting in a low intrinsic porosity (Figure 3d). These building blocks form larger aggregates with some porosity on the higher structural level. This is reflected in the lower DOA absorption number of 1.05 mL/g, as specified by the manufacturer. The structure of the building blocks and their mode of aggregation implies a much higher potential for abrasive action when forcibly moved on a surface. These particles are presumably mechanically much more stable than their counterparts that are used for thickening. In addition, they can take up less fluid and are, thus, less prone to glide over a surface.

### 3.3. Abrasivity of Model Toothpastes Obtained by Profilometric Analysis and RDA

**Figure 4:**
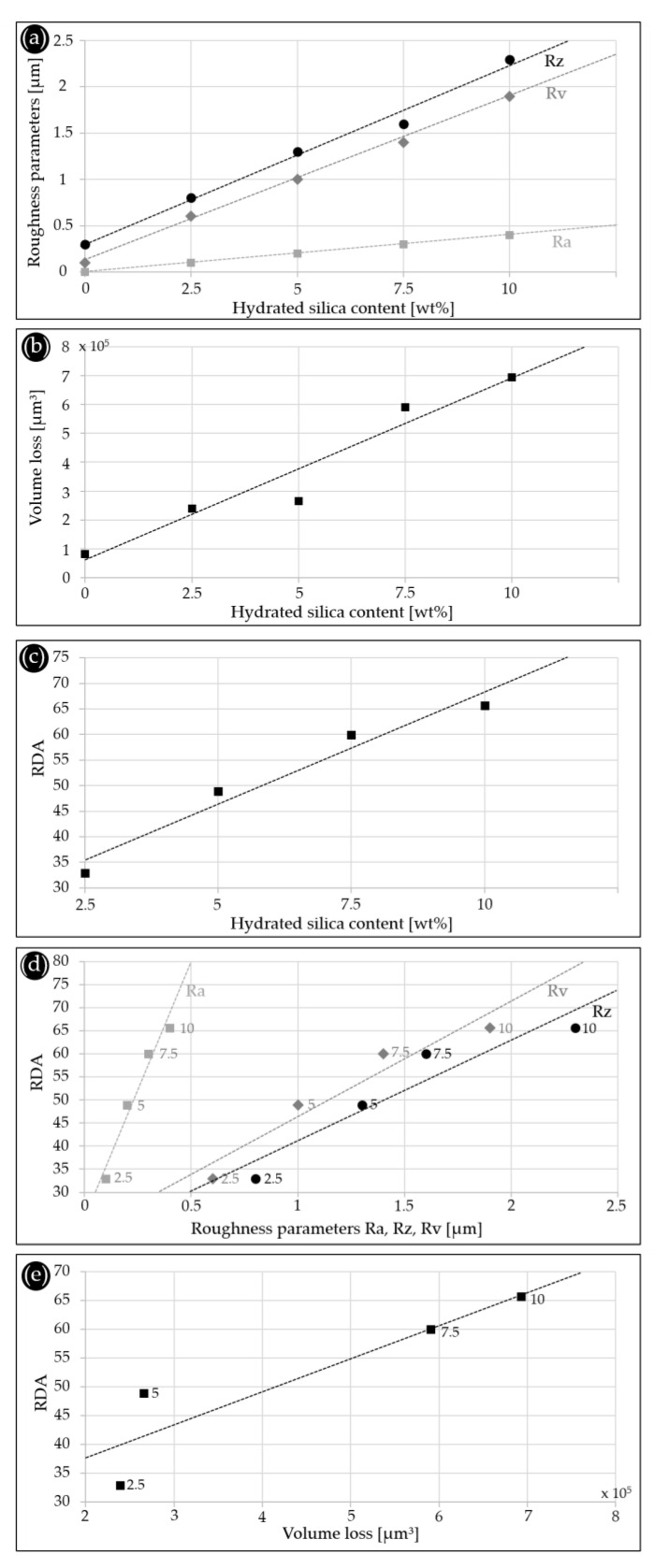
Abrasivity of model toothpaste formulations. (**a**) Mean values (n = 3) of the roughness parameters Ra, Rz and Rv in dependence of the hydrated silica particle content. (**b**) Mean volume loss (n = 3) with increasing weight percentage of hydrated silica, including the control brushed with distilled water only. (**c**) Dependence of RDA on the hydrated silica particle content. (**d**) Correlation between the roughness parameters Ra, Rz and Rv and the RDA values. (**e**) Correlation between the volume loss values and the RDA values of the model toothpastes. The dashed lines in (**a**–**e**) are the corresponding regression lines, the numbers in (**d**,**e**) indicate the hydrated silica content in [wt%].

To compare the results of our study with different approaches presented in the literature [12,17,18], we collected three different roughness parameters (Figure 2c) and the volume loss values. The mean roughness values (Ra, Rz and Rv in µm) and the average volume loss (in µm^3^) of the four model toothpastes with increasing hydrated silica content (2.5, 5.0, 7.5, 10.0 wt%), a reference that was only brushed with distilled water (i.e., without hydrated silica); and the tested commercial toothpastes are given in Table 4. The reference was examined as the control to determine whether the toothbrush itself is capable of damaging the surface of the test specimens. The results show that the mean values for all abrasivity parameters are negligibly small compared to the tests with toothpastes. The corresponding roughness map (Figure 4a) confirms the absence of detectable scratch marks. This is consistent with other tests on PMMA plates [4] which showed that cleaning with a toothbrush and water causes only very little abrasion. However, when toothpaste is added, the toothbrush has been shown to influence the cleaning process. The abrasion values increase up to more than tenfold, depending on the bristle diameter, the number of bristles and the cleaned surface (number of bristles multiplied by the surface area of a single bristle) [4]. Furthermore, it has been shown that the bristle stiffness [23] and terminal shape [24] alone and in combination with different brushing forces [25,26] yield different abrasive wear results on dentine. It is, therefore, crucial to use only one toothbrush model consistently for all tests. Using different toothbrush models may compromise the comparability of results in different studies. Moreover, the abrasive wear on dentine changes for toothpaste slurries with different abrasivity depending on the used brushing force [26]. To avoid such effects and to ensure comparability of our brushing tests with RDA measurements, we chose a brushing force of 1.5 N, according to the standard [10], and kept it constant in all tests.

**Figure 5:**
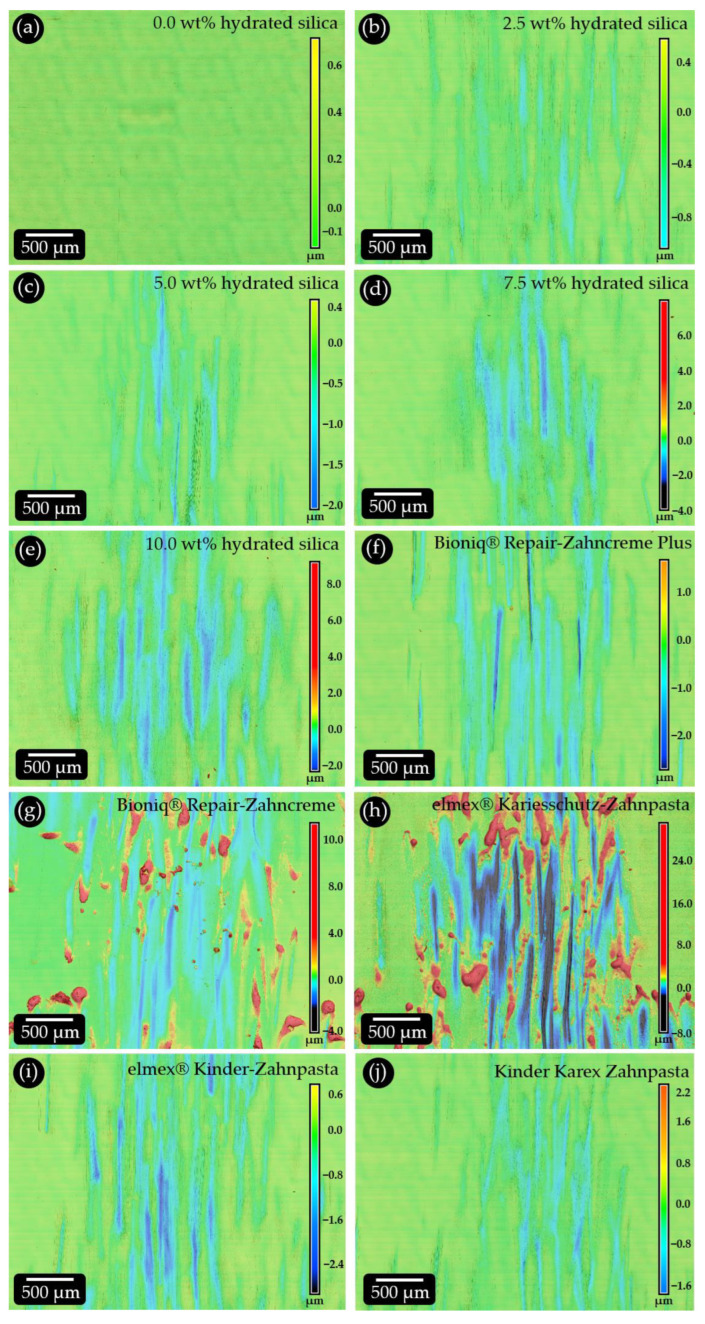
Representative roughness maps obtained by profilometric analysis of the toothbrush simulator abrasion experiments. (**a**) Reference brushed with distilled water. (**b**–**e**) Model toothpaste formulations with (**b**) 2.5 wt%, (**c**) 5 wt%, (**d**) 7.5 wt% and (**e**) 10 wt% of abrasive hydrated silica particles. (**f**–**j**) Samples cleaned with commercially available toothpastes. (**f**) Bioniq^®^ Repair-Zahncreme Plus, (**g**) Bioniq^®^ Repair-Zahncreme, (**h**) elmex^®^ Kariesschutz Zahnpasta, (**i**) elmex^®^ Kinderzahnpasta and (**i**) Kinder Karex Zahnpasta. Since the surface scratch depths varied significantly between the different tested formulations, each map was individually color-coded (see right side of each panel).

For the model toothpastes, the profilometric values (Table 4) of all three roughness parameters (Ra, Rz and Rv) show a linear increase for increasing hydrated silica concentrations (Figure 4a) with very high correlation coefficients. The volume loss values (Table 4) also increase linearly with increasing hydrated silica content (Figure 4b). However, the values varied between the three measured samples per concentration, resulting in a high standard deviation (Table 4). Consequently, the correlation coefficient is slightly lower. Our control samples brushed with distilled water yield results above zero for Rz, Rv and volume loss, showing that the toothbrush has a small influence on the setup, which is generally very sensitive. The representative roughness maps for the different hydrated silica contents confirm the trend (Figure 5). While the number and density of the detected surface scratches remains relatively constant, the depth of the individual scratches increases with increasing abrasive silica concentration (Figure 5b (2.5 wt%), 5c (5.0 wt%), 5d (7.5 wt%), 5d (10.0 wt%)). This results in higher volume losses, which is in good accordance with the literature [27]. Interestingly, the maximum depth of the scratches never exceeds a value of 4 µm, a trend which is independent of the abrasive particle concentration. This depth corresponds to roughly one third of the average particle diameter. A possible explanation is that the particles abrade the sample surface by being horizontally moved by the toothbrush filaments without vertical pressure. If they were “pushed” into the surface, they could probably remove more material. The number of particles present during brushing will consequently influence only the number of scratches. The scratch depth will depend on the size, material and morphology of the particles. Since all filaments of the toothbrush are in contact with the sample surface during brushing, a deeper abrasion can probably be achieved only by increasing the brushing time. This is consistent with observations from other studies that showed that volume loss and scratch depth depend on the combination of toothpaste and toothbrush used [12,28].

The RDA values of the model toothpastes increase with increasing weight percentages of abrasive hydrated silica in the formulations (Figure 4c). A 2.5 wt% hydrated silica content results in the lowest average RDA of 33, followed by 49 (5.0 wt%), 60 (7.5 wt%) and 66 (10.0 wt%) (Table 4). Our data indicate that the RDA does not increase exactly linearly with the content of abrasive silica particles. At high concentrations, the relative increase in RDA is lower than at low abrasive concentrations. This further supports the abrasion mechanism proposed above. Plotting the roughness parameters Ra, Rz and Rv against the measured RDA values results in good correlations with little scatter (Figure 4d, Table 4). When volume loss is plotted against RDA, the regression line shows deviations at low abrasive concentrations (Figure 4d). At 2.5 wt% the RDA is lower, and at 5 wt% abrasive silica values are higher than anticipated by the volume loss values. This correlates with a high scatter in the corresponding volume loss values (Table 4). A possible explanation is that our profilometric analysis is very sensitive. We analyze individual scratches induced by the brushing, which yield different results, especially at low abrasive concentrations. Such size effects are also responsible for the sometimes-high standard deviation values we obtained for the roughness parameters. In addition, surface contaminations can influence the obtained values since the overall volume losses are very small compared to those generated in RDA or RDA-PE measurements. Here, more samples than the three sets analyzed in this study may lead to more reliable results. In summary, all roughness parameters as well as the volume loss values can potentially be used as standard curves for predicting the RDA values achieved by our model toothpastes (Figure 4d,e).

### 3.4. Abrasivity of Commercial Toothpastes Obtained by Profilometric Analysis and RDA

The roughness parameters, the volume loss values and the RDA values obtained for the commercial toothpastes (see Table 3) are given in Table 4. All products use hydrated silica as the main cleaning agent. Kinder Karex Zahnpasta, Bioniq^®^ Repair-Zahncreme Plus and Bioniq^®^ Repair-Zahncreme contain particulate hydroxyapatite, which may also have abrasive properties (Table 3). Bioniq^®^ Repair-Zahncreme also contains tetrapotassium pyrophosphate as an additional abrasive.

**Figure 6:**
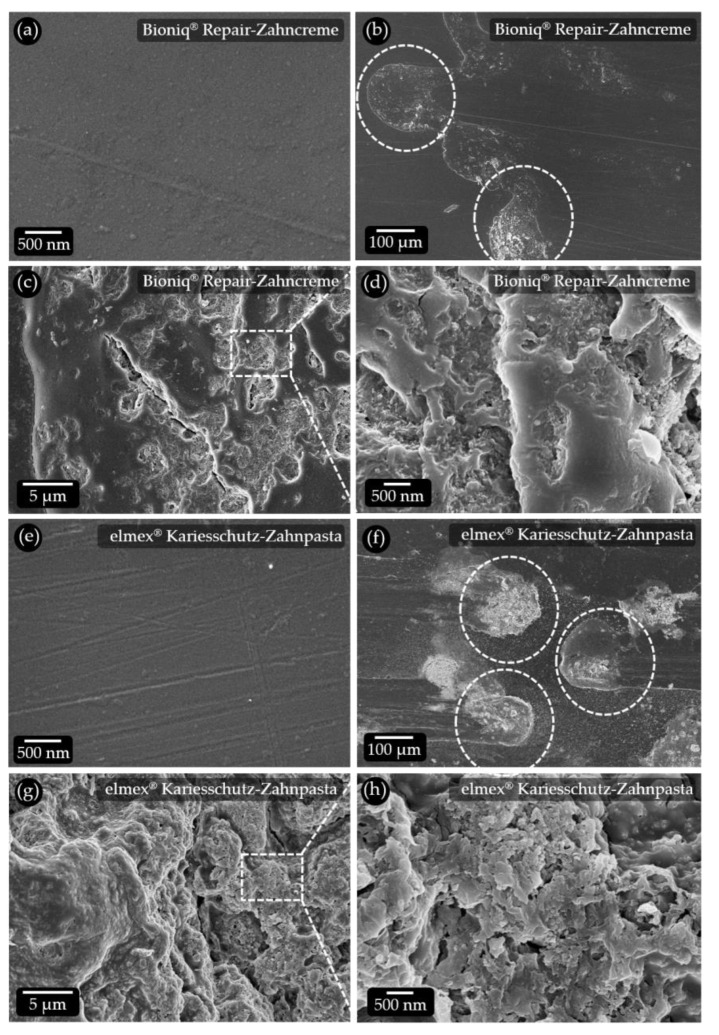
SEM micrographs of the PMMA samples brushed with (**a**–**d**) Bioniq^®^ Repair-Zahncreme and (**e**–**h**) elmex^®^ Kariesschutz-Zahnpasta. Both products show the formation of pile-ups consisting of polymer material and abrasive particles in different degrees of severity.

The lowest RDA of 48 was obtained for Kinder Karex Zahnpasta (Table 4), which is consistent with the low values measured for the roughness parameters and the volume loss. The roughness maps (Figure 5j) confirm this, showing evenly distributed scratches that do not exceed about 2 µm in depth. Bioniq^®^ Repair-Zahncreme has an RDA of 66. However, both Ra and Rz values are higher, and Rv is lower than in toothpastes with higher RDA (Table 4). Possible reasons for this can be found in the corresponding roughness map (Figure 5g). It shows a large number of spots where material has piled up in the shape of spherical deposits located above the reference plane. These are up to 10 µm high, while the scratch depths are generally low. SEM analysis shows that the pristine PMMA surface is smooth (Figure 6a). The pile-ups are located at the ends of the travel paths of individual filaments (Figure 6b). They contain particles that were obviously pushed into the substrate material (Figure 6c). At high magnification, structures resembling the abrasive silica particles embedded in an organic (polymer) matrix can be observed (Figure 6d). A reason for this may be the tetrapotassium pyrophosphate contained in this product, which is known to be abrasive and is one of the standard abrasives used in RDA and RDA-PE tests [10]. A possible mechanism is that the pyrophosphate particles abrade polymer material, which traps the silica particles and piles up at the end of travel of the filaments. Despite its abrasivity, the pyrophosphate content does not increase the abraded volume when compared to Bioniq^®^ Repair-Zahncreme Plus, which does not contain pyrophosphates. In oral care formulations, pyrophosphates are used as a calculus-controlling agent [5]. With a value of 71, the RDA of Bioniq^®^ Repair-Zahncreme Plus is slightly higher than that of Bioniq^®^ Repair-Zahncreme. Ra and Rz are lower, but Rv and volume loss are higher (Table 4). This is reflected in the roughness map (Figure 5f), which shows evenly distributed scratches with depths of up to 3 µm but no obvious traces of pile-ups. elmex^®^ Kinder-Zahnpasta achieves an RDA value of 86. This is surprising since all roughness parameters and the volume loss are lower or equal (Ra) than for Bioniq^®^ Repair-Zahncreme Plus with an RDA of 71. The roughness map (Figure 5i) shows a comparable density of scratches, with a slightly higher number of deeper ones. At present, we are not able to explain this discrepancy. It must be considered that this product may contain ingredients that can have unidentified influences on both RDA and profilometric measurements. The highest RDA (88) was obtained for elmex^®^ Kariesschutz-Zahnpasta (Table 4). Although this value is only slightly higher than the value of 86 obtained for elmex^®^ Kinder-Zahnpasta, the roughness parameter values and the volume loss determined by laser-scan profilometry are much higher. The roughness maps show large areas covered with broad and up to 8 µm deep scratches. Large, irregularly shaped pile-ups that reach over 25 µm in height are present at their ends (Figure 5h). SEM investigation shows that even areas with pristine PMMA are covered with small scratches (Figure 6e). Very large particle pile-ups are located at the ends of filament traces. The filament traces are covered with particles embedded in the sample surface, even where no distinct scratch marks are visible (Figure 6f). Upon closer inspection, the pile-ups show a rugged surface profile (Figure 6g) consisting of organic material intermixed with particles (Figure 6h). These resemble the abrasive hydrated silica particles analyzed in this study (Figure 3c). Compared to all other commercial toothpastes, the abrasion tests with elmex^®^ Kariesschutz-Zahnpasta caused extensive damage to our PMMA surfaces. This cannot be explained by the brushing and abrasive ingredients alone and must, therefore, have other causes. One explanation may be the presence of Olaflur as an active ingredient. Olaflur is an amine fluoride molecule with a long, non-polar hydrocarbon chain that is known to act as a surfactant [29]. Such molecules can infiltrate the PMMA matrix at the surface and act as a plasticizer for the polymer. In consequence, the PMMA sample surface is softened by the long exposure to Olaflur during the abrasion test. Abrasive particles can become implanted into the surface by the toothbrush filaments. The softened superficial polymer layers are then brushed away together with the particles, leaving deep scratch marks, while the removed material forms the pile-ups. A possible reason why this effect was not observed in elmex^®^ Kinder-Zahnpasta could be that this product contains a much lower (one third less) amount of Olaflur, which causes less superficial plasticization of the PMMA. However, the complete absence of pile-ups and deeper scratch marks may be caused by a different particle size and morphology of the used abrasive silica and/or other unknown qualitative and quantitative variations in the formulation. It should also be noted that the effects exerted on PMMA cannot be transferred to the in vivo situation in the oral cavity.

### 3.5. Correlation of Profilometric Analysis and RDA Values

**Figure 7:**
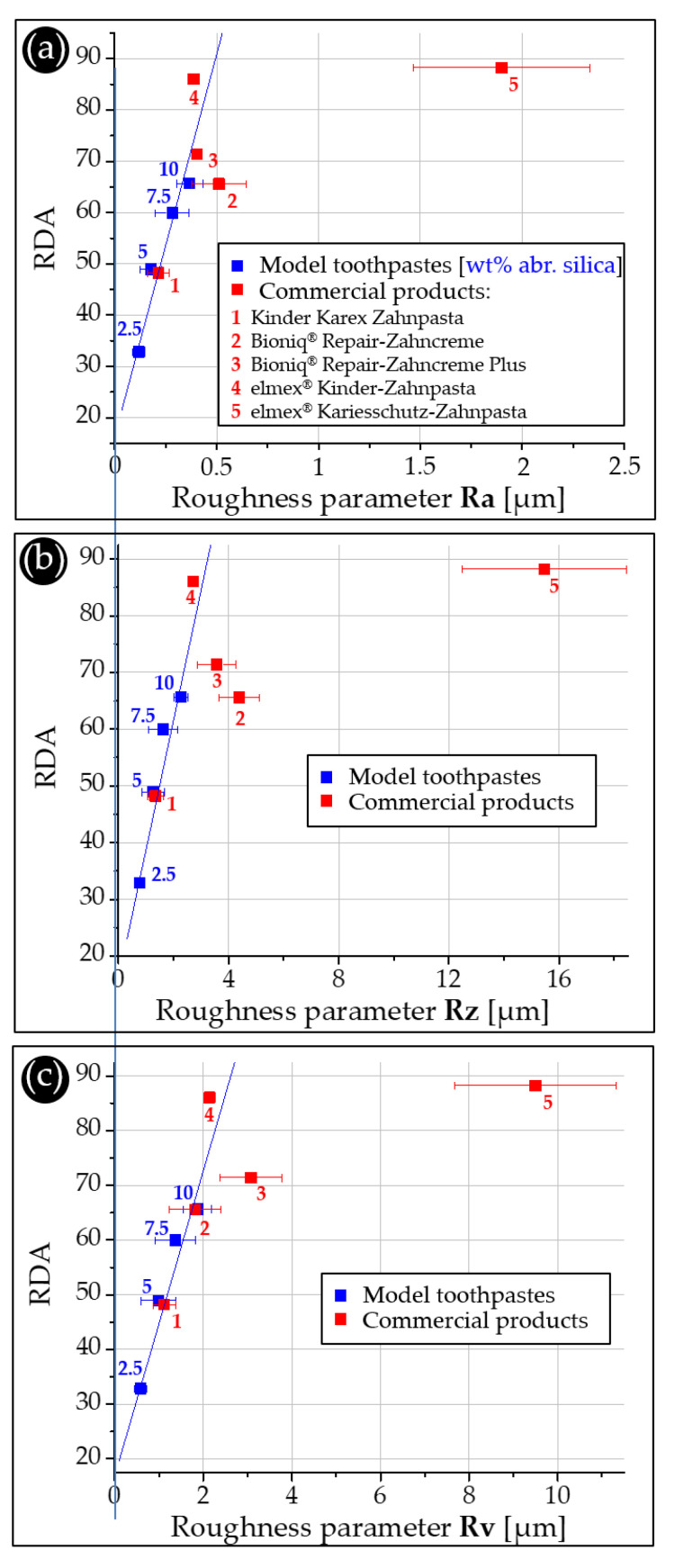
Correlation between the roughness parameters and the associated RDA values. (**a**) Ra values, (**b**) Rz values and (**c**) Rv values of the model toothpastes (blue) and the tested commercial products (red) plotted against their experimentally determined RDA values.

Figure 7 compares the RDA values and the three roughness parameters Ra (Figure 6a), Rz (Figure 6b) and Rv (Figure 6c) by using the respective model toothpaste results as calibration curves. When comparing RDA values with the roughness parameters [14], one must be aware that the standard methods of RDA and RDA-PE consider volume loss only, as quantified against a reference abrasive [10,17,18]. Since Ra considers the mean arithmetic roughness of a sample, both volume loss and volume deposition contribute to the values measured with laser-scan profilometry. Rz is calculated based on the largest distance from the lowest to the highest point with respect to the reference plane. This parameter is, therefore, even more sensitive to pile-ups, as observed for some of our tested commercial toothpastes. Since Rv considers the maximum distance between the reference plane and the deepest point on the surface, it ignores pile-ups. This can lead to inaccurate values, since pile-ups may mask scratches present below them. This is also the case for our volume loss data. Overall, the prediction of RDA values based on our profilometry data is sensitive to mechanistic phenomena occurring on the small (micrometer) scale since they are based on the quantitative analysis of scratch profiles. Nevertheless, the roughness parameters for the commercial toothpastes correlate well with the measured RDA values (Figure 7). An exception is elmex^®^ Kariesschutz-Zahnpasta for the reasons discussed above. Comparable tests on PMMA plates in the past concluded that there is no correlation between the roughness parameters (Ra [12]) and the associated RDA values. However, the RDA values used in that study were based on manufacturer information. This leaves the possibility that they originated from different test institutes and/or different test conditions, such as different toothbrushes and reference abrasives, which may strongly influence the results [4].

**Figure 8:**
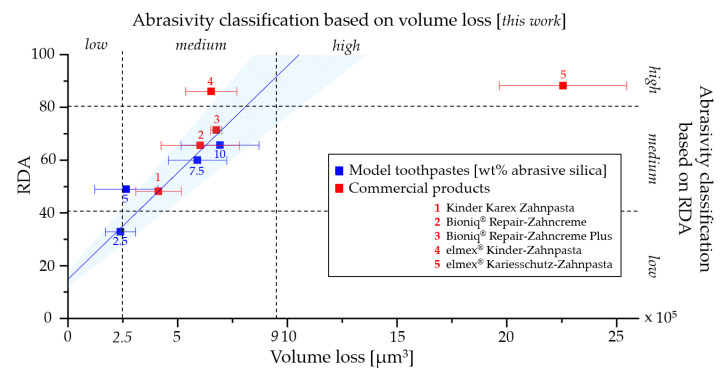
Correlation between measured volume loss and the experimentally determined RDA values obtained for the model toothpastes (blue) and the tested commercial products (red). The model toothpaste regression curve serves as the calibration curve for predicting the RDA values of the commercial products (see Table 3). The area shadowed in blue represents a deviation of 20% in the RDA values above and below the calibration curve [8]. RDA values within this range can be considered to be well predicted by our experimental approach. The dashed lines describe the classification of all tested toothpastes (low, medium and high abrasivity) according to our approach (*x*-axis) and according to an established classification for RDA by Hamza et al. [34].

To verify whether RDA values can be predicted by our profilometric analysis, we chose to use the volume loss values. They are methodically closest to standard RDA and RDA-PE measurements. Figure 8 compares the measured RDA values and the volume loss values using the model toothpaste results as the standard curve. The predicted RDA values were calculated using the formula from the standard curve that was determined by using the model toothpaste values: RDA = 6 × 10^−5^ × volume loss + 26.16 (Table 4, Figure 8). Our results show that the RDA values of our model toothpastes and commercial products can be predicted within a scatter range of 20%, with two exceptions. We consider a deviation of 20% and less as valid, since individual measurements of an RDA test show similar scatter ranges [8]. One exception is elmex^®^ Kariesschutz-Zahnpasta with a much higher predicted RDA value of 162 compared to the 86 determined experimentally. The other is elmex^®^ Kinder-Zahnpasta with a lower predicted RDA of 65 compared to the experimental value of 86. In both cases, the deviations can be explained by possible effects of Olaflur (1400/1000 ppm F^-^) in the formulations. This shows that data can vary because of chemical or physical interactions between the chosen substrate and certain ingredients of toothpaste formulations. Therefore, we conclude that PMMA is a suitable substrate for formulations without ingredients capable of influencing the polymer, but it is not suitable for testing all available toothpaste variants. A possibility to complement our method is, therefore, to include a different, more chemically inert substrate with a similar surface quality. Here, synthetic sintered hydroxyapatite platelets may be a promising alternative for future research [30]. However, certain ingredients of toothpastes, such as biomimetic hydroxyapatite, which is known to have remineralizing effects [31,32,33], could also have surface effects impacting the results.

Based on our experimental results, we propose a classification of abrasivity similar to those existing in the literature based on RDA (e.g., Hamza et. al, 2020 [34], Figure 8). We determine volume loss values between 0 and 2.5 × 10^5^ µm^3^ to be “low”, values between 2.5 × 10^5^ µm^3^ and 9 × 10^5^ µm^3^ to be “medium”, and values above 9 × 10^5^ µm^3^ to be “high” abrasivity. Except for elmex^®^ Kinder-Zahnpasta, the abrasivity classifications (proposed vs. the literature) are in good agreement for our tested commercial toothpastes.

## 4. Conclusions

The in vitro toothbrushing tests with model toothpaste formulations containing increasing amounts of abrasive particles clearly demonstrate that higher concentrations of silica result in higher abrasivity. Comparison with commissioned RDA values of the same formulations shows a good correlation between the roughness parameters (Ra, Rz and Rv) and the volume loss determined by profilometric analysis on PMMA samples. The results show that the RDA can be predicted with high confidence for model toothpaste formulations without specific active ingredients. Investigations of commercial toothpastes, however, show that variations in formulations, especially the presence of ingredients that have the potential to damage PMMA, may lead to different results for profilometric analysis and RDA. Therefore, the proposed testing and evaluation procedure needs to be expanded by using more chemically inert substrate materials. A possible candidate is sintered hydroxyapatite, which offers similar surface quality and, thus, standardized repeatability of the experiments. Extrapolation of RDA values for commercial toothpastes based on the standard curve derived from our model toothpastes shows that only selected products can be predicted. The classification into high, medium and low abrasivity that we propose for our experimental approach correlates well with the established RDA classification (e.g., [32]). Our method determines abrasivity with high resolution by analyzing actual scratch profiles both qualitatively and quantitatively. Thus, mechanistic data such as particle–surface interactions can be derived from the results. These can be combined with other data, such as information on cleaning efficacy [35] in the early stages of toothpaste development. Together with information on abrasivity provided by RDA or RDA-PE, these results help to provide dentists and patients with appropriate toothpastes for specific clinical requirements or individual needs such as tooth whitening or sensitivity relief. Our laser-scan profilometry approach to determine toothpaste abrasivity thus provides a valuable tool for toothpaste developers.

## Figures and Tables

**Table 1:** Ingredients for producing the four model toothpaste formulations and their functions within the formulation (INCI: International Nomenclature Cosmetic Ingredients).

Formulation Ingredients [INCI Denom. (Standard Denom.)/Trade Name (Manufacturer)]	1 [wt%]	2 [wt%]	3 [wt%]	4 [wt%]	Function
Water (Aqua)	57.0	54.6	52.2	49.9	Excipient
**Cellulose Gum** (Sodium carboxymethyl cellulose)/ WALOCEL^™^ CRT 2000 PA Sodium Carboxymethylcellulose (DDP Specialty Products Germany GmbH & Co. KG, Walsrode, Germany)	1.7	1.6	1.5	1.3	Thickening
**Hydrated Silica** (Amorphous silicon dioxide)/ SYLODENT^®^ SM 850C (GRACE GmbH, Worms, Germany)	2.5	5.0	7.5	10.0	Abrasive
**Hydrogenated Starch Hydrolysate** (hydrolyzed starch)/ Meritol 160 Pharma (Tereos Starch & Sweeteners Belgium NV, Aalst, Belgium)	15.0				Humectant, consistency
**Glycerin** (Propan-1,2,3-triol, Glycerol)/ MERCOL^®^ V995 EP RSPO/MB, Non-GMO (cosmetic grade)/GLYCERIN 99.5%, (CREMER OLEO GmbH & Co. KG, Hamburg, Germany)	11.3				Humectant, consistency
**Hydrated Silica** (Amorphous silicon dioxide)/ SYLODENT^®^ SM 880T, (GRACE GmbH, Worms, Germany)	9.5				Thickening
**1,2-Hexanediol** (DL-hexane-1,2-diol), **Caprylyl Glycol** (Octane-1,2-diol)/ SymDiol^®^ 68 (Symrise AG, Holzminden, Germany)	1.0				Preservation, humectants, consistency
**Silica** (Highly dispersed silica, synthetic radio-amorphous silica)/ HDK^®^ N20 PYROGENE KIESELSÄURE HYDROPHIL (Wacker Chemie AG, Munich, Germany)	1.0				Consistency, thickening
**Sodium Methyl Cocoyl Taurate** (Ethanesulfonic acid, 2-(methylamino)-, N-coco acyl derivs., sodium salts)/ ADINOL™ CT95SD-PW-(RB) (Croda Europe Limited, Cowick Hall, Snaith, UK)	1.0				Surfactant, cleaning

**Table 2:** Viscosity and pH values for the four model toothpaste formulations.

Formulation Properties	1	2	3	4
Abrasive hydrated silica content [wt%]	2.5	5.0	7.5	10.0
Dynamic shear viscosity [Pa·s] (n = 3)	45.1 ± 0.6	51.0 ± 2.7	53.0 ± 0.7	56.7 ± 1.2
pH of virgin formulation	6.5	6.4	6.4	6.3
pH of slurry (25 g toothpaste + 40 mL distilled water)	6.9	6.8	6.8	6.8

**Table 3:** Overview of the tested toothpastes, their main active ingredients and the abrasive and potentially abrasive substances.

Toothpaste	Manufacturer	Active Substance	Abrasive
elmex^®^ Kariesschutz-Zahnpasta	CP GABA GmbH, Hamburg, Germany	Olaflur (amine fluoride) (1400 ppm F^−^)	Hydrated silica
elmex^®^ Kinder-Zahnpasta	CP GABA GmbH, Hamburg, Germany	Olaflur (amine fluoride) (1000 ppm F^−^)	Hydrated silica
Bioniq^®^ Repair-Zahncreme Plus	Dr. Kurt Wolff GmbH & Co. KG, Bielefeld, Germany	Hydroxyapatite (20 wt%)	Hydrated silica (5 wt%), Hydroxyapatite (20 wt%)
Bioniq^®^ Repair-Zahncreme	Dr. Kurt Wolff GmbH & Co. KG, Bielefeld, Germany	Hydroxyapatite (20 wt%)	Hydrated silica (5 wt%), Tetrapotassium pyrophosphate, Hydroxyapatite (20 wt%)
Kinder Karex Zahnpasta	Dr. Kurt Wolff GmbH & Co. KG, Bielefeld, Germany	Hydroxyapatite (10 wt%)	Hydrated silica (5 wt%), Hydroxyapatite (10 wt%)

**Table 4:** Roughness parameters Ra, Rz and Rv and volume loss values obtained by profilometric analysis of our abrasion tests with all toothpaste samples, together with the obtained RDA values. The predicted RDA values are based on the volume loss calibration curve calculated from the model toothpaste abrasivity results.

Sampled Formulations	Ra [µm]	Rz [µm]	Rv [µm]	Volume loss [µm^3^]	RDA	Predicted RDA
**Model Toothpastes** Hydrated Silica [wt%]						
0.0	0.0 ± 0.0	0.3 ± 1.9	0.1 ± 0.0	0.8 ×10^5^ ± 0.3 ×10^5^	-	-
2.5	0.1 ± 0.0	0.8 ± 0.2	0.6 ± 0.2	2.4 ×10^5^ ± 0.7×10^5^	33 ± 3	41
5.0	0.2 ± 0.0	1.3 ± 0.4	1.0 ± 0.4	2.7 ×10^5^ ± 1.4 ×10^5^	49 ± 4	42
7.5	0.3 ± 0.1	1.6 ± 0.5	1.4 ± 0.4	5.9 ×10^5^ ± 1.3 ×10^5^	60 ± 6	62
10.0	0.4 ± 0.1	2.3 ± 0.4	1.9 ± 0.4	6.9 ×10^5^ ± 1.8 ×10^5^	66 ± 6	68
**Commercial toothpastes**						
Bioniq^®^ Repair-Zahncreme Plus	0.4 ± 0.0	3.6 ± 0.71	3.1 ± 0.7	6.8 ×10^5^ ± 0.3 ×10^5^	71 ± 9	67
Bioniq^®^ Repair-Zahncreme	0.5 ± 0.2	4.4 ± 1.7	1.8 ± 0.6	6.0 ×10^5^ ± 1.8 ×10^5^	66 ± 4	62
elmex^®^ Kariesschutz-Zahnpasta	1.9 ± 0.5	15.5 ± 5.2	9.5 ± 1.8	22.6 ×10^5^ ± 2.9 ×10^5^	88 ± 17	162
elmex^®^ Kinder-Zahnpasta	0.4 ± 0.1	2.7 ± 0.4	2.1 ± 0.4	6.5 ×10^5^ ± 1.2 ×10^5^	86 ± 9	65
Kinder Karex Zahnpasta	0.2 ± 0.1	1.3 ± 0.4	1.1 ± 0.3	4.1 ×10^5^ ± 1.0 ×10^5^	48 ± 5	51

## Data Availability

Not applicable.
